# Effects of a 12-week training program in outdoor gym equipment in morphological and functional parameters, quality of life, and physical activity levels in older adults

**DOI:** 10.3389/fspor.2024.1444472

**Published:** 2024-09-23

**Authors:** Welmo A. Barbosa, Alexandre F. Machado, Marco Bergamin, Stefano Gobbo, Valentina Bullo, Francisco Luciano Pontes Junior, Alexandre L. Evangelista, Fabiana R. Scartoni, Roberta L. Rica, Danilo S. Bocalini

**Affiliations:** ^1^ Experimental Physiology and Biochemistry Laboratory, Center for Physical Education and Sport of the Federal University of Espirito Santo, Vitoria, Brazil; ^2^ Department of Medicine, University of Padova, Padova, Italy; ^3^ Exercise Physiology and Aging Laboratory-LaFEE, School of Arts, Sciences and Humanities, University of São Paulo, São Paulo, Brazil; ^4^ Department of Physical Education, Ítalo Brasileiro College, São Paulo, Brazil; ^5^ Sport and Exercise Sciences Laboratory, Catholic University of Petrópolis, Petrópolis, RJ, Brazil; ^6^ Department of Physical Education, Estácio de Sá University, Vitoria, Brazil

**Keywords:** older adults, aging, physical exercise, health promotion, popular gym

## Abstract

**Introduction:**

This study aimed to investigate the effectiveness of a supervised physical training program with controlled cadence on functional fitness parameters, quality of life perception, and physical activity level in older adults.

**Methods:**

Sixty physically independent older adults were randomly divided into three groups: Supervised Training (ST, *n* = 20), Unsupervised Training (UT, *n* = 20), and a Control Group (C, *n* = 20). The ST and UT groups participated in a 12-week program, performing exercises three times a week for 30 min. The ST group had structured weekly sessions consisting of a 5-minute warm-up (walking at 60% of max heart rate), 20 sets of 30 s at a moderate pace controlled by a metronome with 30 s of passive recovery, and a 5-minute cool-down on non-consecutive days. The UT group trained spontaneously using senior gym equipment, including elliptical machines, rowing machines, air skiers, and leg press machines. The control group maintained their usual daily routines throughout the study. Parameters evaluated included body mass, body mass index (BMI), muscle thickness (biceps brachii, triceps brachii, and vastus lateralis), and functional capacity tests (walking 10 m [W10 m], rising from a sitting position [RSP], rising from the prone position [RVDP], sitting and rising from a chair and moving around the house [SRCW]). Quality of life was assessed across physical, psychological, environmental, and social domains, and physical activity levels were also measured.

**Results:**

No significant changes (*p* > 0.05) in body mass, BMI, or muscle thickness were observed between groups before and after the intervention. However, significant time effects in functional fitness tests were found only in the ST group for W10 m (*p* = 0.0469), RVDP (*p* < 0.0004), RSP (*p* < 0.0001), and SRCW (*p* < 0.0001). Quality of life improved significantly over time in the ST and UT groups across all domains (*p* < 0.0001). Both ST and UT groups also showed significant increases in weekly physical activity time (*p* > 0.0001).

**Conclusion:**

12 weeks of training improved quality of life perception and physical activity levels in older adults.

## Introduction

Given the rising global population of older adults (1), there's an increasing demand for public policies to enhance their quality of life (2). Alternative fitness programs tailored for older adults have gained attention among these initiatives. These programs strive to foster active lifestyles, thereby mitigating the risk of chronic illnesses (3, 4) and preserving or enhancing functional abilities (5, 6). Nonetheless, accessing free spaces for physical activity is proving to be more challenging, particularly for groups encountering barriers to access and struggling to meet recommended activity levels (7).

Specifically in Brazil, physical activity programs for older adults have been implemented in the last few years (6, 7). Among the available programs, senior gyms are popular (APPIs). These are recognized as outdoor gyms with fitness equipment installed in public places, allowing physical exercise in an organized or spontaneous manner. This kind of activity has been analyzed in some recent studies (8–10). Additionally, the value of these programs in promoting the health of the older Brazilian population is emphasized due to their costless nature, which potentially facilitates access for individuals who have difficulty adhering to a fitness program due to cost (2).

Considering the unique features of senior gyms compared to other programs used in traditional gyms, which tend to have more technologically advanced space and equipment, and their role as a public health promotion policy, it is essential to understand the effectiveness of these types of equipment in long-term physical activity programs. From this perspective, studies have already demonstrated positive results from using this equipment in social and psychological domains (11), levels of physical activity (12), and low incidence of injuries (13). However, studies considering the effectiveness of this type of equipment on physical fitness parameters are still scarce.

To the best of the author's knowledge, only one study by Liu and colleagues (14) investigated the effect of this type of equipment on the functional capacity of older adults. Moreover, based on information from Chow and colleagues (15), users of these spaces did not dedicate sufficient time to achieve general guidelines recommendations. In 2022 (16), we evaluated the psychophysiological repercussions in older adults subjected to different exercise sessions with changes in execution speed in senior gyms. It was demonstrated that different cadences can induce different psychophysiological responses.

Furthermore, the exercise supervision had been associated with improvements on muscle strength, power, functional capacity, and quality of life (17). In addition, direct supervision is more effective in inducing morphological and functional improvements than a low supervision in the older people (18, 19). Studies (17, 20) had been showed that the direct supervision was associated with greater rate of training load and strength gains for both lower- and upper-body muscles.

Thus, given the lack and inconsistent of studies about the mid-long-term exercise effects using senior gym equipment on older adults’ functional capacity parameters, this study aimed to verify the effectiveness of a supervised physical training program on functional fitness parameters, quality of life perception and physical activity time in older adults.

## Material and methods

### Study selection

This study was approved by the Research Ethics Committee of the Federal University of Espirito Santo (4.088.540/2022, CAEE: 29949320.1.0000.5542). Physically independent older adults (> 65 years) were invited to participate voluntarily in this study. The invitation was made in community centers, fitness centers, and posters fixed in the squares and parks near senior gyms. The study sample was considered an intentional non-probabilistic sample, selected for convenience with the experimental design.

The inclusion criteria targeted older adults aged 60 years and above with physical independence. Exclusion criteria were recent hospitalization, symptomatic cardiorespiratory disease, uncontrolled hypertension or metabolic syndrome, severe kidney or liver disease, cognitive impairment or progressive debilitating conditions, obesity impeding physical activity, recent bone fractures, or any other medical contraindications to exercise. Additionally, individuals were excluded if they answered the questionnaire incorrectly, did not meet the minimum 85% attendance rate for the exercise program, did not undergo initial and final evaluations, or failed to provide signed informed consent.

Initially, 80 older adults presented themselves to participate in the project voluntarily, however, after the exclusion criteria application, 60 older adults were considered eligible to participate. The participants were then randomized using a randomization website with blocks of six participants. Each block allocated two participants per group, ensuring balanced recruitment in the study and promoting similarity in initial measurements between groups. According to previous studies (21, 22), this strategy reduces the bias risk and is considered a quality criterion in experimental designs investigating comparison between groups. All participants signed informed consent forms according to the Declaration of Helsinki. After that, they were divided into three groups: Supervised Training (ST, n:20): older adults who underwent a training program with controlled cadence and were supervised by an experience trainer throughout the training program in concordance with previously study (16); Unsupervised Training (UT, n:20): older adults who voluntarily exercised without the supervision of an experience trainer throughout the program; and Control (C, n:20): older adults who did not exercise but received informative support and healthy lifestyle recommendations. To the ST group was performed with a ratio of one trainer for each subject.

### Intervention

All participants performed the activities simultaneously to adapt to the climatic conditions and minimize data contamination from any diurnal differences between groups. All participants underwent familiarization sessions with the equipment to correct gestures and movements that could compromise the exercise during the study and to reduce the risk of accidents or injuries. Only older adults who obtained an 85% minimum frequency in training sessions were included in the analysis.

The older adults in the ST and UT groups performed the exercise sessions in a public square at the same time. The ST and UT groups underwent a 12-week program, with exercises performed 3 times a week for 30 min. The subjects who trained with supervision (ST) underwent weekly 30-minute sessions consisting of five minutes of warm-up (walking at 60% of max heart rate and perceived exertion of five to six according to the BORG CR-10 scale), followed by 20 sets of 30 s using cadence control (one movement every two seconds) monitored by a metronome with 30 s of passive recovery between sets, and five minutes of cool-down (walking at 40% of max heart rate and perceived exertion of four to five according to the BORG CR-10 scale) on non-consecutive days. The UT group was instructed to attend the space spontaneously; however, they trained using only the senior gym equipment proposed by the study in concordance with an actual indication of equipment. Thus, the UT group should be evaluated and allowed to indicate exercise guidelines accurately.

The equipment used to perform the exercises in both (ST and UT) intervention groups was elliptical, rowing, rambler, and leg press machines, as show in Barbosa and colleagues (16). The control group was also instructed to maintain their standard daily routines throughout the investigation.

## Evaluated parameters

### Functional autonomy evaluation

Functional autonomy was evaluated by the Latin American Development Group for Maturity (GDLAM), which our group used in previous studies (23, 24) by a senior evaluator. The GDLAM's protocol was described elsewhere (25) and consists of performing the following tests: walking ten meters (W10 m), rising from the seated position (RSP), rising from the ventral decubitus position (RVDP), and sitting and rising from a chair and walking around the house (SRCW). All tests were performed in the order described above on a single day, using a three-minute interval between tests, providing adequate recovery. The GDLAM's General autonomy functional Index (GI) was calculated according to Dantas and de Souza Vale (25) using the following equation:

GI={[(W10m+RSP+RVDP)×2]+SRCW}/3.

Considering the sum of all points, it was possible to obtain a total score, which was used to classify the older adults’ autonomy into four categories: poor (+28.54 points), regular (28.54–25.25 points), good (25.24 to 22.18 points), and very good (−22.18 points).

### Anthropometric evaluation and muscle thickness

A Cardiomed stadiometer (WCS model, Parana, Brazil) with a 0.1 cm accuracy was used to evaluate height. Body mass was measured using a calibrated Filizola scale (Personal Line 150 Model, São Paulo, Brasil) with a 0.1 kg accuracy. Body mass index (BMI, kg/m^2^) was estimated using the formula: BMI = weight/height^2^. Thus, the following parameters were evaluated: body mass (BM) and body mass index (BMI) according to (23).

Using a portable ultrasound (Bodymetrix, BodyMetrix, BX2000, IntelaMetrix, Inc., Livermore, CA), ultrasonography was used to obtain muscle thickness (MT) measurements of the right side of the body's biceps brachii, triceps brachii, and vastus lateralis muscles, according to previous publications in one-dimensional mode (26, 27). In short, water-soluble transmission gel (Aquasonic 100 Ultrasound Transmission Gel, Parker Laboratories Inc., Fairfield, NJ, USA) was applied at each measurement site, and a 2.5 MHz ultrasound wave was applied to the skin at the measurement site. When the image quality was considered satisfactory, it was saved using appropriate computer technology to obtain muscle thickness dimensions, according to previous publications (26, 27). The sites were measured with a vinyl measuring tape and then marked with a felt-tip pen to ensure measurement accuracy throughout the protocol. During upper extremity measurements, the participants remained seated with their arms relaxed and extended. During lower limb measurements, the individuals remained standing, with body weight distributed in both lower segments. To ensure accuracy, three measures were taken from the segments. A fourth measurement was made if a difference greater than 10% was found. The mean values of the three measurements for each muscle group were used in the analysis.

### Quality of life

Quality of life was assessed by the World Health Organization Quality of Life Assessment (WHOQOL), a questionnaire previously used by our group (28, 29). The questionnaire consists of 25 questions on various aspects of quality of life, including Physical Domain, which concerns pain or discomfort, energy or fatigue, sleep, rest, mobility, dependence on medication, daily activities, and work performance; Psychological Domain, which includes feelings, learning, memory and attention, self-esteem, spirituality, religiosity, and positive or negative thinking; Social Domain, which deals with personal relationships, social support, and sexuality; Environmental Domain, which includes physical safety, home environment, financial security, the opportunity for information assessment, participation in social or cultural events, and activities performed during free time. Each domain was scored from 0 to 100 points; the higher scores represented an improvement.

### Physical activity level

The International Physical Activity Questionnaire Short Form (IPAQ-SF) was applied to assess the level of physical activity. The IPAQ-SF contains questions regarding each physical activity session's weekly frequency and duration for moderate intensity, vigorous, and walking activities. It has been validated previously for older adult populations (30). The level of physical activity was classified based on the frequency and duration of the activity, following the World Health Organization guidelines (31): physically active older adults were those who met the minimum recommendation of 150 min of physical activity per week, whereas physically inactive older adults are those who do not meet the minimum recommendation of 150 min of physical activity per week.

### Statistical analysis

The sample size required was estimated using G* Power 3.1 software (version 3.1.9.4). The power analysis assumed an estimating error of α + 0.05 and power = 80% to an actual power of 0.80. The Shapiro-Wilk test was used to assess normality. Two factors ANOVA for repeated measures were used to evaluate the effect of time and intervention: time (pre- and post-intervention) vs. groups (ST, UT, and C) with *post hoc* Bonferroni when appropriate. The effect size was estimated using Cohen's (27). For this, the following interpretation was used: small (d = 0.2), medium (d = 0.5), and large (d ≥ 0.8). All statistical analyses were performed using GraphPad Prism software (version 4.0, San Diego, CA, USA) with a significance level of *p* < 0.05, with data presented as mean ± standard deviation.

## Results

**Figure 1:**
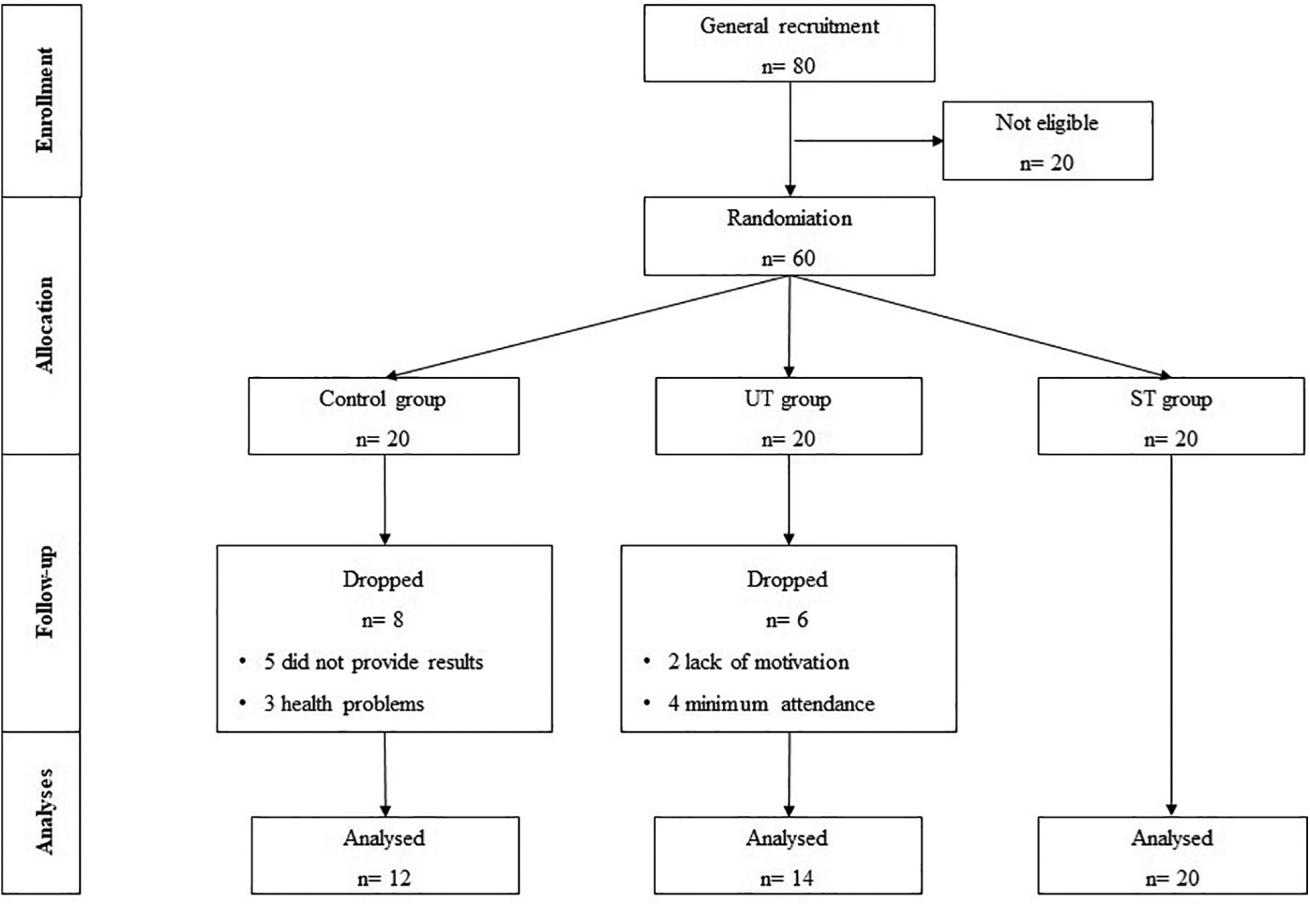
Flow chart of the study intervention. Control group (**C**), unsupervised training (UT), and supervised training (ST).

As shown in Figure 1, five older adults from the control group did not provide results during the intervention period, and three indicated health problems that prevented the second evaluation. Regarding the UT group, 14 individuals completed the intervention period, with two individuals reporting a lack of motivation and dropping out of the project, and a further four individuals did not meet the minimum attendance requirement. Regarding the ST group, no changes in the number of subjects were identified during the intervention period.

### Effects of 12 weeks intervention on functional parameters

Table 1 shows no significant differences (*p* > 0.05) between groups before the intervention. Significant effects over time for the W10 m (F = 4.185, *p* = 0.0469), RVDP (F = 14.90, *p* < 0.0004), RSP (F = 17.56, *p* < 0.0001), SRCW (F = 51.40, *p* < 0.0001), and IG (F = 50.51, *p* < 0.0001) parameters were reported, as well as effects on the interaction of all parameters, as shown in Table 1. After 12 weeks of training, all values in the ST group's functional parameters differed (*p* < 0.05) from all other groups. No differences were found between UT and C (*p* > 0.05).

**Table 1:** Effect of 12 weeks of training using senior gym equipment in functional parameters of older adults.

Parameters	Before	After	MD	95% CI	ES	ANOVA		
						Time	Time × group	
						*P*	F	*P*
W10m								
ST	6.90 ± 2.31	5.40 ± 1.81*^,^^†^	1.50	1.013 to 1.987	0.64	< 0.0001		
UT	6.71 ± 1.43	7.07 ± 1.26	0.36	−0.939 to 0.225	0.25	= 0.4014	25.20	< 0.0001
C	7.00 ± 1.70	7.33 ± 1.96	0.33	−0.962 to 0.295	0.19	= 0.5810		
RSP								
ST	11.45 ± 4.35	8.60 ± 3.03*^,^^†^	2.85	2.051 to 3.649	0.65	< 0.0001		
UT	11.57 ± 2.84	11.50 ± 2.56	0.07	−0.883 to 1.026	0.02	> 0.9999	25.18	< 0.0001
C	11.25 ± 1.65	11.66 ± 1.49	0.41	−1.448 to 0.614	0.24	= 0.9591		
RVDP								
ST	5.05 ± 1.05	3.80 ± 0.83*^,^^†^	1.25	0.837 to 1.663	1.19	< 0.0001		
UT	4.92 ± 1.07	4.85 ± 1.40	0.07	−0.421 to 0.564	0.06	> 0.9999	14.18	< 0.0001
C	5.25 ± 1.05	5.16 ± 1.02	0.08	−0.449 to 0.616	0.08	> 0.9999		
SRCW								
ST	43.95 ± 18.17	29.10 ± 1.31*^,^^†^	14.85	12.000 to 17.700	0.81	< 0.0001		
UT	44.14 ± 13.13	41.07 ± 12.97	3.07	−0.332 to 6.476	0.23	= 0.0893	43.64	< 0.0001
C	43.66 ± 10.10	45.00 ± 9.58	1.33	−5.010 to 2.344	0.13	> 0.9999		
GI								
ST	30.15 ± 10.35	21.50 ± 6.80*^,^^†^	8.65	7.119 to 10.180	0.83	< 0.0001		
UT	30.28 ± 5.44	29.42 ± 5.45	0.85	−0.973 to 2.687	0.15	= 0.7493	54.99	< 0.0001
C	30.25 ± 5.04	30.91 ± 4.60	0.66	−2.644 to 1.310	0.13	> 0.9999		

Values are expressed as the mean, standard deviation of the control group (C), unsupervised training (UT), and supervised training (ST) of walking 10 m (W10 m), rising from a sitting position (RSP), rising from the prone position (RVDP), sitting and rising from the chair and move around the house (SRCW) and GI, GDLAM's index; MD, mean difference; 95% CI, 95% of the confidence interval; ES, effect size.

* *p* < 0.05 vs. C.

† *p* < 0.05 vs. UT.

### Effects of 12 weeks of intervention on anthropometric parameters

As shown in Table 2, no significant effect was found on time on body mass (F = 0.0097, *p* = 0.9220), height (F = 1.072, *p* = 0.3062), and BMI (F = 0.0775, *p* > 0.7819) around groups.

**Table 2:** Effects of 12 weeks of training using senior gym equipment to anthropometric parameters in older adults.

Parameters	Before	After	MD	95% CI	ES	ANOVA		
						Time	Time × group	
						*p*	F	*p*
Body mass (kg)								
ST	70.54 ± 10.39	70.77 ± 9.84	0.23	−1.024 to 0.557	0.2	> 0.9999		
UT	73.64 ± 10.85	73.62 ± 10.79	0.10	−0.718 to 0.746	0.0	> 0.9999	0.5043	= 0.6075
C	72.62 ± 10.13	72.45 ± 9.91	0.17	−0.442 to 0.782	0.1	> 0.9999		
Height (m)								
ST	1.60 ± 0.08	1.61 ± 0.09	0.00	−0.012 to 0.008	0.0	> 0.9999		
UT	1.66 ± 0.14	1.65 ± 0.15	0.00	−0.001 to 0.018	0.0	> 0.1052	1.944	> 0.1554
C	1.60 ± 0.09	1.60 ± 0.09	0.00	−0.008 to 0.008	0.0	> 0.9999		
BMI (kg/m^2^)								
ST	27.31 ± 3.84	27.29 ± 3.40	0.02	−0.286 to 0.337	0.0	> 0.9999		
UT	28.04 ± 5.01	28.07 ± 4.93	0.3	−0.321 to 0.256	0.0	> 0.9999	0.1899	> 0.8277
C	28.19 ± 4.38	28.13 ± 4.33	0.6	−0.180 to 0.302	0.1	> 0.9999		

Values are expressed as the mean, standard deviation of the control group (C), unsupervised training (UT), and supervised training (ST) of body mass, height and body mass index (BMI). MD, mean difference; 95% CI, 95% of the confidence interval; ES, effect size.

### Effects of 12 weeks of intervention on muscle thickness

No significant effect was found over time for the muscle thickness of the biceps brachii (F = 0.8712, *p* > 0.3558), triceps brachii (F = 0.05088, *p* > 0.8226), and vastus lateralis (F = 0.0629, *p* = 0.8032), as shown in Table 3.

**Table 3:** Effects of 12 weeks of training using senior gym equipment to muscular thickness parameters in older adults.

Parameters	Before	After	MD	95% CI	ES	ANOVA		
						Time	Time × group	
						*p*	F	*p*
Biceps brachii (mm)								
ST	22.08 ± 1.88	22.16 ± 1.69	0.08	−0.517 to 0.350	0.04	> 0.9999		
UT	22.21 ± 1.96	22.28 ± 1.85	0.07	−0.473 to 0.330	0.03	> 0.9999	0.0095	> 0.9905
C	21.55 ± 2.45	21.65 ± 2.53	0.10	−0.436 to 0.236	0.04	> 0.9999		
Triceps brachii (mm)								
ST	18.58 ± 2.84	18.58 ± 2.60	0.00	−0.553 to 0.553	0.00	> 0.9999		
UT	17.57 ± 3.45	17.50 ± 2.98	0.07	−0.441 to 0.583	0.02	> 0.9999	0.3666	> 0.6952
C	16.00 ± 2.69	16.15 ± 2.70	0.15	−0.578 to 0.278	0.05	> 0.9999		
Vastus lateralis (mm)								
ST	15.41 ± 1.78	15.50 ± 1.78	0.08	−0.475 to 0.309	0.05	> 0.9999		
UT	15.07 ± 1.73	15.00 ± 1.66	0.07	−0.291 to 0.434	0.04	> 0.9999	0.3075	> 0.7369
C	14.70 ± 2.31	14.75 ± 2.40	0.05	−0.353 to 0.253	0.02	> 0.9999		

Values are expressed as the mean, standard deviation of the control group (C), unsupervised training (UT), and supervised training (ST) of muscle thickness. MD, mean difference; 95% CI, 95% of the confidence interval; ES, effect size.

There were no significant differences regarding percentage variation between groups for the biceps brachii (C: 1.00 ± 1.65%, UT: 1.64 ± 2.17%, ST: 1.65 ± 1.92%; F = 0.4936; *p* = 0.6138) and triceps brachii (C: 2.00 ± 2.33%, UT: 2.85 ± 1.65%, ST: 2.00 ± 1.91%; F = 0.9283; *p* = 0.4030) muscles. After the intervention period, the percentage change in thickness of the vastus lateralis muscle was similar between the ST (3.30 ± 2.59%) and UT (2.28 ± 2.40%) groups (*p* > 0.05). However, the ST group had significantly higher values (F = 4.965; *p* = 0.0115) than the C group (0.75 ± 0.86%), which did not differ from the UT group.

### Effects of 12 weeks intervention on the perception of quality of life

Table 4 presents the values related to the perception of quality of life. Similarly to the functional data, no significant differences were identified before the intervention. Significant effects over time on the physical (F = 78.45; *p* < 0.0001), psychological (F = 101.6; *p* < 0.0001), social (F = 54.60; *p* < 0.0001), and environmental (F = 21.68; *p* < 0.0001) domains were found, with a significant effect on interaction for all parameters.

**Table 4:** Effects of 12 weeks of training using senior gym equipment to perceive quality-of-life parameters in older adults.

Parameters	Before	After	MD	95% CI	ES	ANOVA		
						Time	Time × group	
						*p*	F	*p*
Physical								
ST	61.65 ± 8.64	71.70 ± 5.94*^,^^†^	10.05	−11.99 to −8.114	1.31	< 0.0001		
UT	61.28 ± 8.40	64.00 ± 7.65	2.71	−5.028 to −0.400	0.32	= 0.0166	30.96	< 0.0001
C	61.16 ± 7.72	62.33 ± 6.59	1.16	−3.666 to 1.333	0.15	= 0.7540		
Psychological								
ST	61.40 ± 8.96	70.95 ± 6.09*	9.55	−12.44 to −6.655	1.06	< 0.0001		
UT	58.28 ± 8.46	71.85 ± 9.25*	13.57	−17.03 to −10.11	1.60	< 0.0001	20.99	< 0.0001
C	56.58 ± 6.54	57.16 ± 5.85	0.58	−4.321 to 3.154	0.08	> 0.9999		
Environmental								
ST	59.55 ± 10.29	68.45 ± 8.08*	8.90	−13.91 to −3.887	0.86	= 0.0002		
UT	58.64 ± 10.55	68.28 ± 14.16*	9.64	−15.63 to −3.651	0.91	= 0.0007	4.259	< 0.0205
C	54.33 ± 9.36	54.75 ± 9.16	0.41	−6.888 to −6.055	0.04	> 0.9999		
Social								
ST	58.60 ± 7.40	67.85 ± 5.86*	9.25	−12.58 to −5.924	1.25	< 0.0001		
UT	59.14 ± 10.23	69.35 ± 6.27*	10.21	−14.19 to −6.239	0.99	< 0.0001	10.51	< 0.0002
C	58.33 ± 10.78	58.83 ± 10.13	0.50	−4.794 to 3.794	0.04	> 0.9999		

Values expressed as the mean standard deviation of the control (C), unsupervised training (UT), and supervised training (ST) groups on the parameters of physical, psychological, social, and environmental domains. MD, mean difference; 95% CI, 95% of the confidence interval; ES, effect size.

* *p* < 0.05 vs. C.

† *p* < 0.05 vs. ST.

After 12 weeks of using the intervention program, there were no significant differences in the psychological, social, and environmental domains of perception and quality of life between the ST and UT groups (*p* > 0.05). However, the ST and UT groups significantly differed in the physical domain (*p* < 0.05). Additionally, the values of the perception and quality of life domains of group C were lower (*p* < 0.05) compared to the ST and UT groups.

### Effects of 12 weeks of intervention on weekly time of physical activity

**Figure 2:**
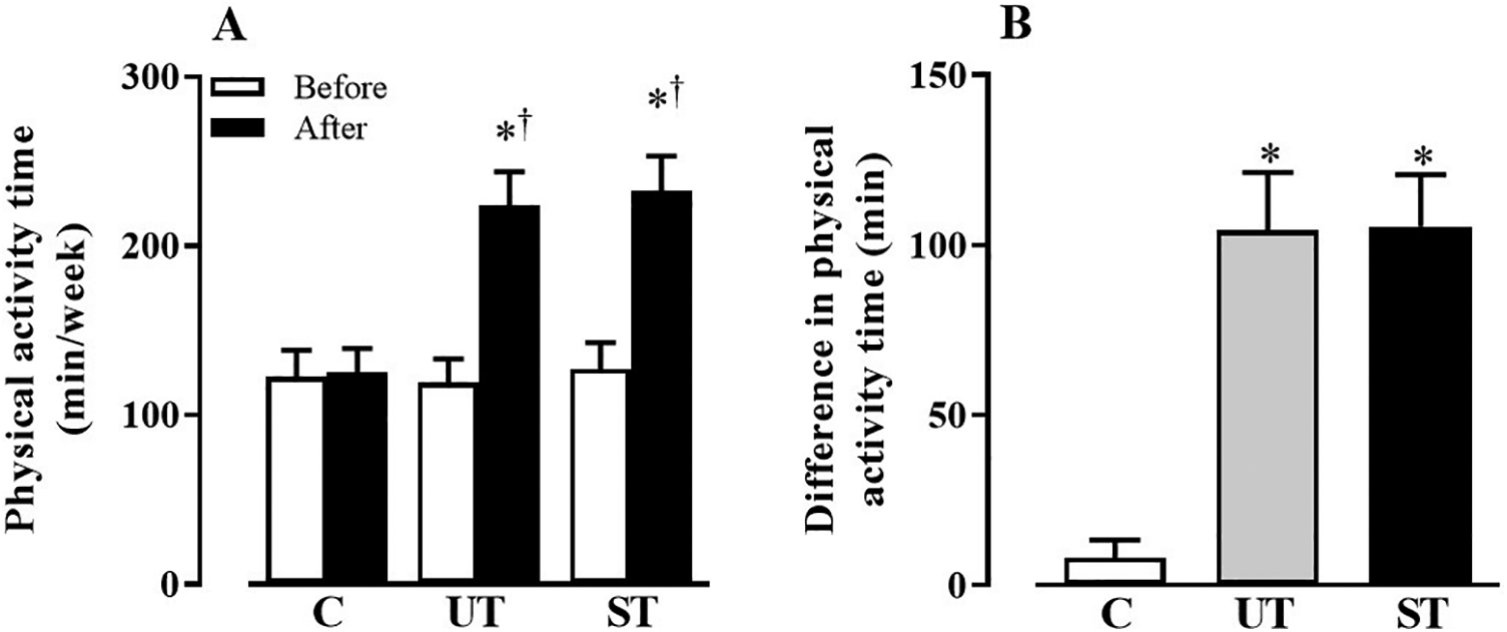
Values expressed as the mean standard deviation of the control (**C**), unsupervised training (UT), and supervised training (ST) groups of the time of physical activity **(A)** and the difference in the time of physical activity **(B)** before and after the intervention. *p < 0.05 vs. C. ^†^*p* < 0.05 vs. before.

Figure 2A presents the amount of time older adults spent on physical activity each week, not including the 30 min of intervention. The results showed a significant effect over time (F = 1,030, *p* > 0.0001) and in the interaction (F = 215, *p* > 0.0001).

Unlike C group (Before: 122.41 ± 15.92 min, After: 125.41 ± 13.89 min, MD[95% CI]: −3.00[13.54–7.53], ES: 0.15, *p* > 0.9999), which did not present changes after the intervention period, significant increases in the time of physical activity were found in the ST (Before: 127.00 ± 15.88 min, After: 232.50 ± 20.74 min, MD[95% CI]: −105.5[−113.7 to −97.34], ES: 6.64, *p* < 0.0001) and UT (Before: 119.21 ± 13.79 min, After: 223.57 ± 20.23 min, MD[95% CI]: −3.00[13.54–7.53], ES: 104.4[−114.1 to −94.60], ES: 7.56, *p* < 0.0001) groups, which did not differ from each other, as shown in Figure 2A. Figure 2B shows the difference in time before and after the intervention period. The values of ST (105.50 ± 15.37 min) and UT (104.40 ± 17.04 min) groups, although similar, differed (F = 209.4; *p* < 0.0001) from C (8.00 ± 5.29 min).

## Discussion

This study aimed to verify the effectiveness of a supervised and unsupervised physical training program on functional capacity parameters, perception of quality of life, and physical activity time for older adults. The key findings revealed that only supervised training (ST) significantly improved functional capacity, while both supervised (ST) and unsupervised training (UT) positively impacted quality of life perception and physical activity levels. With aging, individuals often experience a decline in functional capacity, making daily tasks more challenging. Physical fitness, encompassing strength, endurance, flexibility, agility, balance, and coordination, becomes crucial for maintaining independence and health in older adults.

Parallel to this scenario, functionality in older people has often been considered a vital component in Geriatrics and Gerontology. The functional capacity can be defined as the efficiency of older adults in meeting the physical demands of daily life, which range from basic activities for independent living to more complex actions contained in daily routines. Therefore, actions aimed at autonomy and independence are essential for the individual to perform basic and instrumental daily activities satisfactorily. In addition, it is known that regular physical exercise after the age of 50, as well as the adoption of an active lifestyle, have positive impacts on health and longevity, contributing to the improvement of functional capacity and, consequently, allowing older adults to be independent in making decisions and contributing to their lives and daily routines. Additionally, we believe that evaluating functional independence is essential for numerous actions, such as the progression of physical training and monitoring independence in performing daily activities.

In this study, functional autonomy evaluation utilized the test battery of GDLAM (25), revealing significant improvements only in the ST group. The supervised training offered advantages over unsupervised exercise practice. The study suggests that supervisioned training induces more significant skeletal muscle overload, leading to potential neural and metabolic adjustments that positively impact functional response. To our knowledge, only one study by Liu and colleagues (14) investigated the effect of using senior gym equipment on functional capacity in older adults, showing no efficacy of its use after 12 weeks of training. Therefore, it is possible to consider that supervised intervention indicated a considerable advantage compared to unsupervised exercise practice.

Considering supervised exercise practice, studies conducted with different modalities, such as water aerobics (28, 32), strength training (33), functional exercises (34), and multicomponent training (23), demonstrated efficacy in improving the functional capacity of older adults. One hypothesis to explain our findings is that the supervised exercise, compared to unsupervised practice, induces greater skeletal muscle overload, with possibilities for different neural and metabolic adjustments. Considering this, classic studies have shown that the association of more excellent muscular activity, promoted by greater recruitment of motor units, as well as metabolic changes, induced by greater intensity in task performance, can promote morpho-functional modifications in skeletal muscle, impacting on the functional response. Additionally, we did not control the number of movements in the UT group. However, the number of movements performed per session was lower, impacting lower volumes of repetitions and possibly less muscular activity intensification. In the literature, the knowledge about the influence of the number of repetitions, the magnitude of muscular activity given by the intensity of the training session, and the number of exercises are classic parameters that can significantly influence the neuromuscular response, both in strength development and in the structure of the muscular segment (35).

In addition, based on the findings by Barbosa and colleagues (16), which found no significant changes in perception or enjoyment during sessions with a similar cadence to that used in our study (although this variable was not assessed in our research), it is possible to speculate that the ST group may have been more engaged throughout the program and training compared to the UT group. Thus, negative experiences can negatively impact exercise adherence, suggesting that this point could also be considered a reason for disengagement since greater engagement in physical activity programs is associated with better outcomes. However, further studies should be conducted to clarify these findings.

Interestingly, changes in body mass and muscle thickness were not observed in the ST or UT groups. The absence of significant alterations in these anthropometric parameters may be attributed to factors such as caloric intake and the use of body weight as the primary training overload. It is essential to highlight that monitoring caloric intake in exercise programs is critical. In our study, although the maintenance of dietary habits was indicated, caloric intake was not controlled during the exercise program. This fact has been indicated as a significant limiting factor for investigating exercise-induced morphological changes. Therefore, we assume that the results found in the anthropometric parameters of the present study should be carefully employed, and any generalization should be avoided.

Considering that muscle thickness is an anthropometric parameter and used as an indicator of muscular hypertrophy (21, 22), in our study, we did not identify changes in all analyzed muscular segments after the intervention period. Although the mechanisms for understanding hypertrophy are still intensely debated, mechanical stress has been considered the main trigger for increased myofibrillar synthesis and, consequently, hypertrophy (36). Additionally, higher recruitment of muscle fibers can maximize energy expenditure and metabolic stress, stimulating anabolic and intracellular signaling pathways and increasing protein synthesis. In our study, the overload used in the program was only body weight, and unfortunately, it was not possible to consider the activation of muscle fibers and metabolic characteristics during the exercises used.

Considering the use of bodyweight exercises to improve muscle hypertrophy, the literature is still inconclusive, and further studies are warranted. While some have shown a positive effect (37), others have found no significant impact (27) of bodyweight exercises on hypertrophy in programs that utilize high intensity in young individuals. Finally, in the same way that caloric intake can interfere with anthropometric parameters, the same can be applied to muscle thickness since it is already known that protein intake can influence the maintenance or increase of fat-free mass as well as body composition of both adults (38), and older adults (39).

The study also emphasized the subjective and multidimensional nature of the concept of quality of life. While physical activity is recognized as essential for enhancing the quality of life in older adults, the prevalence of physical inactivity remains a significant public health concern. Creating open public spaces equipped with exercise facilities, such as senior gyms, offers a sustainable approach to promoting physical activity among the general population.

From this perspective, constructing environments like open public spaces in parks or squares equipped with exercise equipment is a sustainable approach to boosting physical activity levels among the general population. This has already become a frequent practice in other countries (40, 41). These spaces are effective in promoting improvements in the perception of well-being and also provide a method to facilitate the accessibility and democratization of physical activity in different populations. Our study demonstrated that interventions performed with and without supervision effectively improved older adults’ perception of quality of life (Table 2). This result confirms the findings of previous studies (8, 13), which revealed that both the practice of physical activity, supervised and unsupervised, can improve older adults’ perception and quality of life.

Our findings agree with Santana and collegues (41) that discuss the need for the presence of physical education professionals in these environments to enhance, by the appropriate use of these devices, the execution of the movements by the user, aiming at improving older adults’ quality of life. Based on our findings, the presence of physical education professionals is not necessary for improving quality of life. In addition, the indication that using this equipment and practicing physical activity in these spaces tends to be considered risky remains inconsistent. According to Silva and colleagues (13), the occurrence of injuries during the practice of physical activity in senior gyms was 3.4%, showing that the use of equipment for the practice of physical activities is safe for the physical integrity of users, regardless of gender and age group.

However, although the presence of physical education professionals in these spaces is a subject of debate, we consider that the presence of these professionals may not guarantee greater effectiveness of the practice itself, especially given the presence of informative signs about the use of equipment in these locations. There is no consensus about devices since they do not require a complex procedure to increase intensity/load and can be easily adjusted to fit various body dimensions. Thus, it is necessary to take a broader look at the presence and performance of the physical education professional in these spaces. The use of strategies based simply on the prescription of exercises can be an inefficient strategy. Therefore, the association of educational and behavioral strategies can be a viable alternative for including professionals in these spaces, mainly due to their effectiveness (10). Additionally, modifying the equipment structure to allow adjustments to suit better the participants’ conditioning and implementing structures or devices (such as a metronome) to increase the workload are of great value and deserve attention.

It should be mentioned that older adults in this study had low physical activity levels and were considered insufficiently active. However, we demonstrated that regular practice, both supervised and unsupervised, allowed an increase in the level of physical activity among older adults. Studies (3, 6) demonstrated that physical activity can effectively increase physical activity spontaneously, especially considering leisure activities. Considering the changes in the level of physical activity, the interventions (ST and UT) in the present study were not effective in reaching the classical recommendations for physical activity for older adults, despite the program consisting of 90 min, distributed over three weekly days, with physical activity intensity considered moderate.

Both supervised and unsupervised interventions effectively improved older adults’ perception of quality of life, challenging the necessity of physical education professionals in these environments. The study emphasized that using senior gym equipment and physical activity in such spaces can be safe and beneficial for older adults (13). Despite the low initial physical activity levels among older adults, both supervised and unsupervised programs successfully increased their physical activity levels.

Some limitations should be considered, such as sample composed by men, the low level of physical activity among older adults, the intervention time, the monitoring/control of calorie intake, functional and clinical measures do not allow for generalizations. Although the evaluation of muscle thickness is a strategy performed in older adults, in the present study, the measurements were one-dimensional. Moreover, the images obtained in older adults do not have the same quality as those obtained in young individuals. Therefore, the data regarding muscle thickness should be carefully considered. Finally, senior gyms present a considerable variety of equipment, so our results should not be applied to other exercise/equipment or session designs, which does not allow for the generalization of our findings.

In conclusion, a 12-week training program using senior gym equipment improved older adults’ quality of life and physical activity levels. Supervised training was particularly advantageous for improving functional capacity. Further research is recommended to validate these findings, explore training progression possibilities, and assess the influence of different equipment and session designs on older adults’ outcomes.

## Data Availability

The raw data supporting the conclusions of this article will be made available by the authors under reasonable request.

## References

[1] IBGE, I. IBGE divulga as estimativas populacionais dos municípios para 2017. noticias/releases/16131-ibge-divulga-as-estimativas-populacionais-dos-municipios-para2017.html. Published 2017. Assessado em janeiro de 2020 (2017).

[2] Harris ERA Resende HGD Porto F Silva NSLD. Motivos da adesão de idosos às academias da terceira idade. Rev Bras Geriatr Gerontol. (2020) 23:e200117. 10.1590/1981-22562020023.200117

[3] Silva PSCD Boing AF. Fatores associados à prática de atividade física no lazer: análise dos brasileiros com doenças crônicas. Ciência & Saúde Coletiva. (2021) 26:5727–38. 10.1590/1413-812320212611.32432020 34852104

[4] Saint-Maurice PF Coughlan D Kelly SP Keadle SK Cook MB Carlson SA Association of leisure-time physical activity across the adult life course with all-cause and cause-specific mortality. JAMA Netw Open. (2019) 2(3):e190355–e190355. 10.1001/jamanetworkopen.2019.0355 30848809 PMC6484624

[5] de Salles BF Senna GW Furtado HL Simão R. Efeitos do programa de exercícios físicos, das academias da terceira idade, sobre a composição corporal e capacidade funcional de idosos. Rev Andal Med Deport. (2020) 13(1):10–5. Available online at: https://dialnet.unirioja.es/servlet/articulo?codigo=7670842

[6] de Macedo RM de Oliveira MDRP Cilião MR Prosdócimo ACG de Macedo ACB França D Nível de atividade física de idosos participantes de um programa de prevenção de doença cardiovascular. ASSOBRAFIR Ciência. (2019) 6(3):10–20. 10.47066/2177-9333/ac.20901

[7] Sá GBARD Dornelles GC Cruz KG Amorim RCDA Andrade SSCDA Oliveira TP O programa academia da saúde como estratégia de promoção da saúde e modos de vida saudáveis: cenário nacional de implementação. Cien Saude Colet. (2016) 21:1849–60. 10.1590/1413-81232015216.09562016 27276540

[8] Pinheiro WL Coelho Filho JM. Perfil dos idosos usuários das academias ao ar livre para a terceira idade. Rev Bras Promoç Saúde. (2017) 30:1. 10.5020/18061230.2017.p93

[9] de Salles BF Senna GW Furtado HL Simão R. Perfil da aptidão física de idosos ingressantes nas academias da terceira idade. ConScientiae Saúde. (2017) 16(3):318–26. 10.5585/conssaude.v16n3.7644

[10] Costa B Freitas C Silva K. Atividade física e uso de equipamentos entre usuários de duas academias ao ar livre. Rev Bras Ativ Fis Saúde. (2016) 21(1):29–38. 10.12820/rbafs.v.21n1p29-38

[11] Mathias NG Melo J Szkudlarek AC Gallo LH Fermino RC Gomes ARS. Motivos para a prática de atividades físicas em uma academia ao ar livre de paranaguá-PR. Rev Bras Cienc Esporte. (2019) 41:222–8. 10.1016/j.rbce.2018.03.030

[12] Cranney L Phongsavan P Kariuki M Stride V Scott A Hua M Impact of an outdoor gym on park users’ physical activity: a natural experiment. Health Place. (2016) 37:26–34. 10.1016/j.healthplace.2015.11.002 26699448

[13] Silva ATD Fermino RC Alberico CO Reis RS. Fatores associados à ocorrência de lesões durante a prática de atividade física em academias ao ar livre. Rev Bras Med Esporte. (2016) 22:267–71. 10.1590/1517-869220162204151226

[14] Liu YC Yang WW Fang IY Pan HLL Chen WH Liu C. Training program with outdoor fitness equipment in parks offers no substantial benefits for functional fitness in active seniors: a randomized controlled trial. J Aging Phys Act. (2020) 28(6):828–35. 10.1123/japa.2019-0009 32470918

[15] Chow HW Mowen AJ Wu GL. Who is using outdoor fitness equipment, and how? The case of Xihu park. Int J Environ Res Public Health. (2017) 14(4):448. 10.3390/ijerph14040448 28430141 PMC5409648

[16] Barbosa WA Rica RL Pontes Junior FL Reis VM Bergamin M Bocalini DS. Psychophysiological effects of different execution speeds of single about exercise in outdoor fitness equipment performed by older adults. Mot Rev Educ Fis. (2022) 28:1–8. 10.1590/S1980-657420220020521

[17] Mazzetti SA Kraemer WJ Volek JS Duncan ND Ratamess NA Gomez AL The influence of direct supervision of resistance training on strength performance. Med Sci Sports Exerc. (2000) 32(6):1175–84. 10.1097/00005768-200006000-00023 10862549

[18] Ramírez-Campillo R Martínez C de La Fuente CI Cadore EL Marques MC Nakamura FY High-speed resistance training in older women: the role of supervision. J Aging Phys Act. (2017) 25(1):1–9. 10.1123/japa.2015-0122 27182680

[19] Vieira DCL Nascimento DC Tajra V Teixeira TG Farias DL Tibana RA High supervised resistance training in elderly women: the role of supervision ratio. Int J Exerc Sci. (2020) 13(3):597–606. 32509119 10.70252/WNVF6463 PMC7241618

[20] Gentil P Botarro M. Influence of supervision ratio on muscle adaptations to resistance training in nontrained subjects. J Strength Cond Res. (2010) 24(3):639–43. 10.1519/JSC.0b013e3181ad3373 19661830

[21] Yudkin PL Stratton IM. How to deal with regression to the mean in intervention studies. Lancet. (1996) 347(8996):241–4. 10.1016/S0140-6736(96)90410-9 8551887

[22] Barnett AG Van Der Pols JC Dobson AJ. Regression to the mean: what it is and how to deal with it. Int J Epidemiol. (2005) 34(1):215–20. 10.1093/ije/dyh299 15333621

[23] Suzuki FS Evangelista AL Teixeira CVLS Paunksnis MRR Rica RL Evangelista RAGDT Effects of a multicomponent exercise program on the functional fitness in elderly women. Rev Bras Med Esporte. (2018) 24:36–9. 10.1590/1517-869220182401179669

[24] Guimaraes SM da Silva CG Rica RL Maia AF Alonso AC João GA Morphofunctional characterization of elderly women according to depressive symptomatology. Manual therapy. Posturology Rehabil J. (2019):1–5. 10.17784/mtprehabjournal.2019.17.669

[25] Dantas EHM de Souza Vale RG. Protocolo GDLAM de avaliação da autonomia funcional. Fitness Perf J. (2004) 3(3):175–82. 10.3900/fpj.3.3.175.p

[26] Brigatto FA Soares EG Braz TV Decamargo J Hartz B Batista CS Acute effect of different duration of foam rolling protocols on muscle thickness, pain pressure threshold, and volume load on multiple sets of knee extension. Int J Exerc Sci. (2021) 14(3):742. 34567358 10.70252/RSHS5778 PMC8439700

[27] Evangelista AL Teixeira CLS Machado AF Pereira PE Rica RL Bocalini DS. Effects of a short-term whole-body, high-intensity, intermittent training program on morphofunctional parameters. J Bodyw Mov Ther. (2019) 23(3):456–60. 10.1016/j.jbmt.2019.01.013 31563355

[28] Bocalini DS Serra AJ Rica RL Santos LD. Repercussions of training and detraining by water-based exercise on functional fitness and quality of life: a short-term follow-up in healthy older women. Clinics. (2010) 65:1305–9. 10.1590/S1807-59322010001200013 21340219 PMC3020341

[29] Rica RL Shimojo GL Gomes MC Alonso AC Pitta RM Santa-Rosa FA Effects of a kinect-based physical training program on body composition, functional fitness, and depression in institutionalized older adults. Geriatr Gerontol Int. (2020) 20(3):195–200. 10.1111/ggi.13857 31923924

[30] Benedetti TRB Antunes PDC Rodriguez-Añez CR Mazo GZ Petroski ÉL. Reproducibility and validity of the international physical activity questionnaire (IPAQ) in elderly men. Rev Bras Med Esporte. (2007) 13:11–6. 10.1590/S1517-86922007000100004

[31] World Health Organization. WHO guidelines on physical activity and sedentary behavior. Geneva: World Health Organization (2020).

[32] de Castro JBP de Oliveira Brum RD Pernambuco CS de Souza Vale RG. Análise de correlação entre força muscular, IGF-1 e autonomia funcional em mulheres idosas com excesso de peso sujeitas a exercício aquático resistido. Rev Invest Activid Acuá. (2019) 3(5):18–23. 10.21134/riaa.v3i5.157538

[33] Borba-Pinheiro CJ Dantas EHM de Souza Vale RG Drigo AJ de Alencar Carvalho MCG Tonini T Resistance training programs on bone related variables and functional independence of postmenopausal women in pharmacological treatment: a randomized controlled trial. Arch Gerontol Geriatr. (2016) 65:36–44. 10.1016/j.archger.2016.02.010 26956618

[34] de Matos DG Mazini Filho ML Moreira OC de Oliveira CE de Oliveira Venturini GR Da Silva-Grigoletto ME Effects of eight weeks of functional training in the functional autonomy of elderly women: a pilot study. J Sports Med Phys Fitness. (2016) 57(3):272–7. 10.23736/S0022-4707.16.06514-2 27441915

[35] Garber CE Blissmer B Deschenes MR Franklin BA Lamonte MJ Lee IM American College of sports medicine position stand. Quantity and quality of exercise for developing and maintaining cardiorespiratory, musculoskeletal, and neuromotor fitness in apparently healthy adults: guidance for prescribing exercise. Med Sci Sports Exerc. (2011) 43(7):1334–59. 10.1249/MSS.0b013e318213fefb 21694556

[36] Schiaffinoa S Reggianib C Akimotod T Blaauw B. Molecular mechanisms of skeletal muscle hypertrophy. J Neuromuscul Dis. (2021) 8:169–83. 10.3233/JND-200568 33216041 PMC8075408

[37] Moghaddam M Estrada CA Baghurst T Jacobson BH. Muscular morphological adaptations of two whole-body high-intensity interval training configurations. J Sports Med Phys Fitness. (2020) 60(7):985–91. 10.23736/S0022-4707.20.10526-7 32343081

[38] Antonio J Peacock CA Ellerbroek A Fromhoff B Silver T. The effects of consuming a high protein diet (4.4 g/kg/d) on body composition in resistance-trained individuals. J Int Soc Sports Nutr. (2014) 11(1):19. 10.1186/1550-2783-11-19 24834017 PMC4022420

[39] AmamouTNormandinEPouliotJDionneIJBrochuMRiescoE. Effect of a high-protein energy-restricted diet combined with resistance training on metabolic profile in older individuals with metabolic impairments. J Nutr Health Aging. (2017) 21:67–74. 10.1007/s12603-016-0760-827999852

[40] Feng Y Liu E Yue Z Zhang Q Han T. The evolutionary trends of health behaviors in Chinese elderly and the influencing factors of these trends: 2005–2014. Int J Environ Res Public Health. (2019) 16(10):1687. 10.3390/ijerph16101687 31091753 PMC6571823

[41] Santana SAV dos Santos Carvalho A Moioli A Venturini ACR Júnior JRG de Araújo RG Presença de profissionais de educação física em academias ao ar livre frequentadas na terceira idade. Rev CPAQV. (2021) 13(3):2.

